# Shaping viral immunotherapy towards cancer-targeted immunological cell death

**DOI:** 10.3389/fonc.2025.1540397

**Published:** 2025-07-08

**Authors:** Anastasia S. Isaeva, Adriana D. Trujillo Yeriomenko, Esther Idota, Sofya I. Volodina, Natalia O. Porozova, Evgeny E. Bezsonov, Alexander S. Malogolovkin

**Affiliations:** ^1^ Molecular Virology laboratory, First Moscow State Medical University (Sechenov University), Moscow, Russia; ^2^ Moscow Institute of Physics and Technology, Dolgoprudny, Russia

**Keywords:** oncolytics, cell targeting, immunotherapy, virus engineering, pseudotyping, retargeting, ICD, TIME

## Abstract

**Background:**

Oncolytic viruses (OVs) have the ability to efficiently enter, replicate within, and destroy cancer cells. This capacity to selectively target cancer cells while inducing long-term anti-tumor immune responses, makes OVs a promising tool for next-generation cancer therapy. Immunogenic cell death (ICD) induced by OVs initiates the cancer-immunity cycle (CIC) and plays a critical role in activating and reshaping anti-cancer immunity. Genetic engineering, including arming OVs with cancer cell-specific binders and immunostimulatory molecules, further enhances immune responses at various stages of the CIC, improving the specificity and safety of virotherapy.

The aim of this study is to update current knowledge in immunotherapy using OVs and to highlight the remarkable plasticity of viruses in shaping the tumor immune microenvironment, which may facilitate anti-cancer treatment through various approaches.

**Methodology:**

Research articles, meta-analyses, and systematic reviews were retrieved from PubMed, using the search terms (‘Oncolytics’ OR ‘Immunotherapy’ OR ‘Virotherapy’ OR ‘Viral vector’) AND ‘gene therapy’, without language restrictions.

**Results:**

In this review, we discuss current strategies aimed at increasing the tumor specificity of OVs and improving their safety. We summarize and functionally categorize different biochemical approaches, with a focus on virus engineering and advancements in immunotherapy. Transduction targeting methods (e.g., xenotype switching, pseudotyping, cell receptor targeting) and non-transduction modifications (e.g., miRNA, optogenetics, transcriptional targeting) are critically reviewed. We also examine the mechanisms of ICD and viral modifications that contribute to efficient cancer cell death and modulation of cancer-specific immunity. Finally, we provide an outlook on promising future oncolytics and approaches with potential therapeutic benefit for the next generation of cancer immunotherapy.

**Conclusion:**

Immunogenic cell death induced by oncolytic viruses is a key mediator of potent anti-cancer immunity. The genetic integration of immunostimulatory molecules as regulatory elements into OV genomes significantly enhances their therapeutic potential, safety, and stability. Additionally, therapeutic potency can be further increased by deleting viral genes that inhibit apoptosis, thereby enhancing ICD. However, the synergistic effects of these modifications may vary significantly depending on the cancer type.

## 1 Introduction

Translational cancer research is among the fastest-growing fields in next-generation medicine, focusing on the development and application of innovative therapeutic strategies to target malignant cells. Despite recent advances in cancer treatment, several types of malignant tumors continue to exhibit resistance to conventional chemotherapy and radiotherapy (1). Coupled with aggressive phenotypes and, in some cases, inaccessibility for surgical removal, cancer remains a leading cause of mortality, accounting for nearly 10 million deaths annually worldwide. The incidence of cancer varies widely depending on cancer type, gender, and, in some instances, racial disparities. According to the American Cancer Society, the cancer mortality rate in the United States has continued to decline, with a 2% annual decrease from 2016 to 2020, largely attributable to advancements in modern cancer therapies and diagnostics. Nevertheless, the overall incidence of cancer continues to rise. In 2022, over 19.9 million new cases were diagnosed globally, with the highest incidence rates observed in lung, female breast, colorectal, prostate, and stomach cancers. Projections estimate that by 2040, there will be more than 28.4 million new cancer cases worldwide. This alarming trend underscores the urgent need for the development of novel, cancer-specific, safe, and highly effective therapeutics (2, 3).

Immunotherapeutic approaches have significantly enhanced our understanding of the role of the tumor microenvironment in cancer progression and therapeutic resistance. It is now well established that activating or stimulating the patient’s immune system through external interventions is crucial for effectively eliminating cancer cells and preventing their dissemination (4, 5).

Oncolytic viruses (OVs) are a cutting-edge form of immunotherapy. They are based on attenuated or recombinant human or animal viruses that selectively infect cancer cells due to the impaired antiviral defenses often found in tumors, leaving normal cells largely unharmed. OVs have key features that clearly articulate their mode of action (6).

1. Direct oncolysis of cancer cells (e.g. Adenoviruses (Ads) and Herpes simplex viruses (HSVs) and releasing tumor-associated antigens (TAAs) — a process that is critical for initiating immunogenic cell death (ICD) (6).
2. Release of DAMPs (Damage-Associated Molecular Patterns): DAMPs such as ATP, HMGB1, and calreticulin are vital for ICD. These molecules act as signals that enhance dendritic cell (DC) maturation and antigen presentation, leading to a stronger antitumor immune response (7). Oncolytic viruses like reoviruses and Newcastle disease virus (NDV) have been engineered to enhance the release of these DAMPs, thereby amplifying ICD. Recent studies have shown that modifications to increase the release of DAMPs or enhance their signaling can significantly improve the efficacy of oncolytic viruses (6).
3. ICD enhancement: promoting the secretion of proinflammatory cytokines such as IL-12 and GM-CSF (granulocyte-macrophage colony-stimulating factor). These modifications help to shift the tumor microenvironment from an immunosuppressive (“cold”) state to an immunostimulatory (“hot”) state, thus facilitating a more effective immune response (8).
4. ICD modulation by synergy with immune checkpoint inhibitors (ICIs): combining ICIs (e.g. anti-PD-1 or anti-CTLA-4), with oncolytic viruses would enhance immune checkpoint development. While ICIs aid in overcoming immune resistance sometimes observed with monotherapy, oncolytic viruses cause direct oncolysis and the release of DAMPs (9). Current clinical trials have shown that this combination approach can result in better patient outcomes and more antitumor responses (10, 11).

Furthermore, OVs can effectively regulate the expression of immune checkpoint molecules, effectively rendering tumor cells susceptible to cytotoxic anti-cancer therapy (12). In addition, due to the relatively simple and modular structure of their genomes, viruses are frequently used in gene therapy to transport effector molecules to cells and tissues. Combinatorial strategies involving oncolytic viruses, immune checkpoint inhibitors (e.g., PD-1, CTLA-4, LAG-3), immunostimulatory molecules (e.g., interferons such as IFN-γ and IFN-β), TAAS, or tumor-specific antigens (TSA) (e.g., MAGE-3, claudin 18.2, mesothelin, E6, E7), as well as chemotherapy agents like the kinase inhibitor Sorafenib, aim to enhance anti-cancer efficacy and overcome the limitations associated with monotherapy (13, 14). While immune checkpoint inhibitors and CAR T-cell therapies have shown promise, they are also associated with certain limitations and adverse effects. In contrast, oncolytic viruses represent a promising therapeutic modality that combines the benefits of both approaches. They have the potential to induce immunogenic cell death (ICD) and activate tumor-specific immune responses with minimal severe toxic effects (15, 16).

In this review, we describe strategies proposed in translational cancer research to enhance the anti-cancer activity of oncolytic viruses, improve tumor specificity, regulate gene expression, and increase safety. We summarize the mechanisms of action of oncolytic viruses and reveal the complex relationship between OVs and the tumor microenvironment. We broke down and clustered the major approaches that have been used for oncolytics in preclinical and clinical settings. Research articles, meta-analyses, and systematic reviews were retrieved from PubMed, using the search terms (‘Oncolytics’ OR ‘Immunotherapy’ OR ‘Virotherapy’ OR ‘Viral vector’) AND ‘gene therapy’, without language restrictions. The retrieved research was categorized according to the mechanism of action, with particular emphasis on immune response modulation. The main targeting approaches are critically discussed, and the potential of oncolytic virotherapy as a strategy for cancer immunotherapy is summarized.

## 2 Oncolytic viruses induce immunogenic cancer cell death rewiring anti-cancer immunity

ICD is a unique form of regulated cell death that arises due to pathological changes in the intracellular or extracellular environment. As a result, dying cells release DAMPs or alarmins into the extracellular space or display them on their surface. ICD activation requires reactive oxygen species (ROS) and endoplasmic reticulum stress (15). Dudek et al. categorized ICD inducers into two types based on their effects on the endoplasmic reticulum (ER). Type I inducers target processes not directly related to the ER, whereas type II inducers lead to cell death by directly inducing endoplasmic stress. Oncolytic viruses are categorized as type II inducers, particularly because they can induce ER stress through excessive viral protein synthesis (15).

Importantly, in addition to its antitumor role, the immune system can stimulate tumor growth through signaling with certain cytokines (IL-1β, IL-23, IL-11, IL-6, TNF, and GM-CSF). Thus, understanding how ICD inducers can bypass this signaling is crucial (17–20).

**Figure 1:**
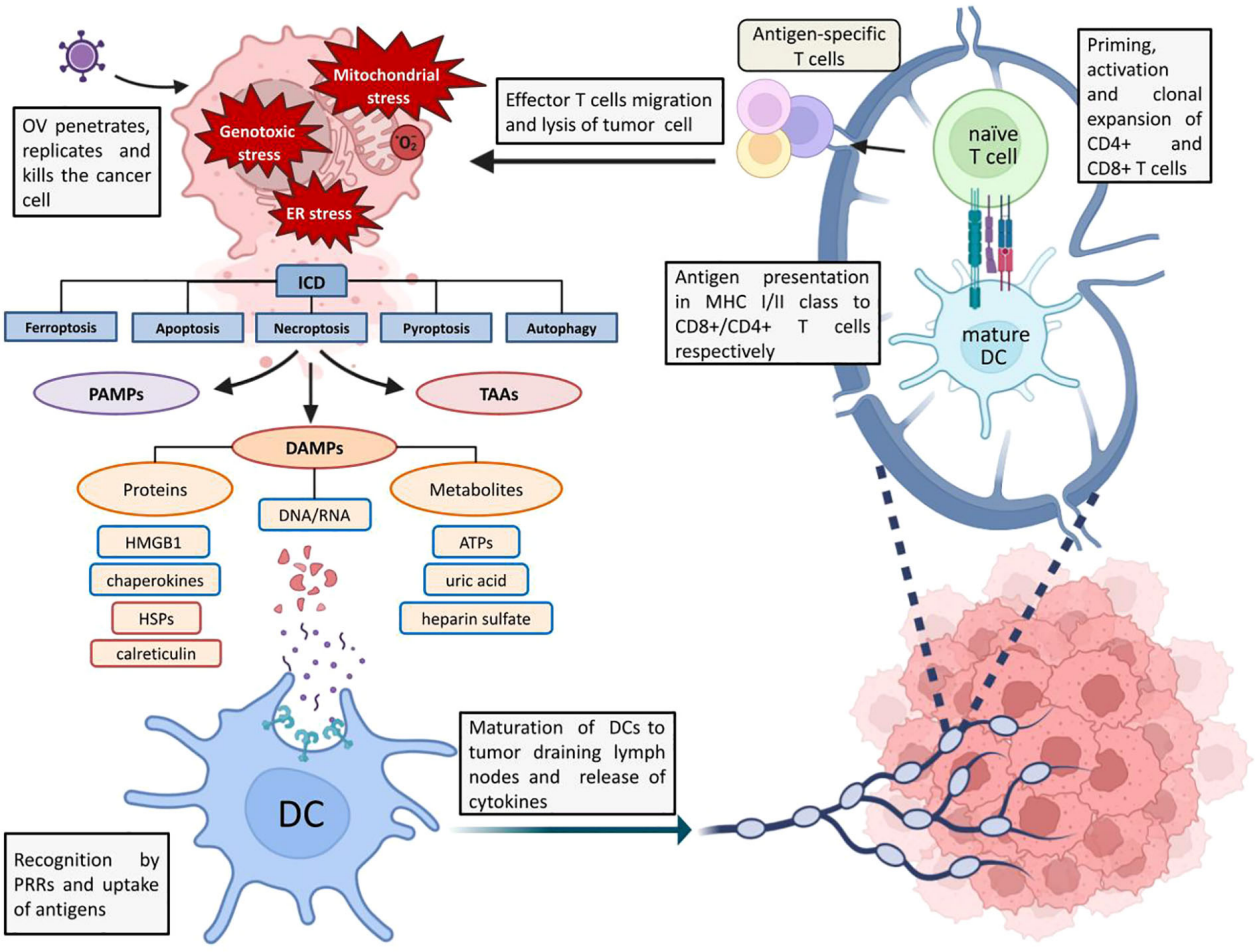
Schematic representation of ICD and OV-mediated antitumor response. ER stress, increased ROS levels and genotoxic stress caused by OV infection trigger ICD. DAMPs, PAMPs and TAAs are released from the dying cell into the extracellular environment, which are recognized by antigen-presenting cells (in particular these are BATF 3+ DCs (dendritic cells), which are involved in viral clearance and antitumor response) via innate immunity receptors (in particular TLRs), captured and processed (22). The binding of DAMPs, PAMPs and TAAs to TLRs triggers the process of DCs maturation and, in order to avoid the emergence of tolerance to tumor antigens and to implement the antitumor T-cell response, at this stage there is a release of proinflammatory cytokines (IL-1β, IL-6, IL-12, TNF (22)), chemokines (IL-8, MCP1) by DCs and other signaling molecules activating innate lymphoid cells (21). After migration to tumor-draining lymph nodes, mature DCs present antigens to CD4+ and CD8+ T cells as part of MHCII and MHCI, respectively (22). Mature DCs have increased expression of MHCI and MHCII as well as co-stimulatory molecules (CD40, CD80, etc.). At this stage there is a transition from innate to adaptive immunity: priming and activation of antigen-specific effector T cells followed by clonal development of T cells in tumor draining lymph nodes and migration to the tumor under the action of various chemokines (CXCL9, CXCL10), where recognition and lysis of cancer cells by T cells occurs (21).

In addition to DAMPs, TAA, and TSA, pathogen-associated molecular patterns (PAMPs) stimulate the immune system during OV-induced ICD. In 2013, Chen and Mellman (21) introduced the concept of Cancer-Immunity Cycle (CIC), describing the principle of immune system activation in response to cancer cell death and demonstrated how its action can be enhanced at each stage of the cycle. **Figure 1** illustrates its interpretation in the context of oncolytic virus action on tumors.

## 3 Tumor immune microenvironment

The relationship between OVs, tumor, and immune system cannot be fully understood without considering the tumor microenvironment, which mediates the interaction between tumor and healthy tissues. The tumor microenvironment is composed of cellular components (such as immune cells, fibroblasts, and blood vessel endothelial cells), extracellular matrix, and various signaling molecules (cytokines and chemokines) (23). An important component of the tumor microenvironment is cancer stem cells (CSCs), which are capable of self-renewal and direct oncogenesis. Targeting CSCs is one of the promising strategies for cancer treatment (24). The immune components of tumor microenvironment also referred to as TIME, include lymphocytes, granulocytes, and macrophages (23). The latter are the most abundant and play a role in tumor development by promoting the entry (escape) of tumor cells into the circulating system and suppressing antitumor immunity (23). Based on their activity, immune cells could be divided into tumor-antagonizing (effector T cells, NK cells, dendritic cells, M1-polarized macrophages, and N1-polarized neutrophils) and tumor-promoting immune cells (regulatory T cells (Tregs), myeloid-derived suppressor cells (MDSCs), and N2-polarized neutrophils). B cells have a dual mode of action (e.g., Bregs) (25). Effector T cells include CD8+ cytotoxic T cells (CTL) and CD4+ T helper cells. The major immune cell types, their roles within the TIME and the agents that activate or suppress them are summarized in **Table 1**.

**Table 1:** Immune cells in TIME.

Cell type	Mechanism	Activation	Suppression	Reference
CD8+ T cells (CTLs)	- Killing cancer cells by granule exocytosis and apoptosis stimulated by death ligands in the cells such as TRAIL-FasL - Induction of cytotoxicity through the release of IFN-γ and TNFα - IFN-γ production induces M1 polarity of macrophages and release of chemokines to attract CD4+ T cells. - Release of TNFα promotes anti-M2 polarization of macrophages	-Cross-presentation of MHC class I antigens by DCs to CD8+ T-cells induces the generation of CTLs. -Ligand interactions on DCs CD70 and CD80-CD86) and receptors on CD8+ T cells (CD27 and CD28) play a key role in priming CD8 + T cells.-Leaded by cytokines (CXCL9 and CXCL10) secreted by DCs CTLs migrate into the tumor. -IFN-γ stimulates the production of CTLs -Promotion by CD4+ T-cells -Stimulatory checkpoints (CD40L, etc.)	- Tregs, MDSCs and cancer cells -Adenosine released by cancer cells stimulates Tregs and MDSCs mediated suppression -By the action of immunosuppressive mediators (IDO1, PD-L1, COX-2, STAT3) released by cancer cells - By the action of TNF-α, TGF-β, IL-6. -Inhibitory checkpoints (CTLA-4, PD-1, etc.)	(26, 27)
CD4+ T helper cells	- Interaction with MHC class II antigens and activation of CD8+ T cells - Optimization of the quality and magnitude of CTLs responses by priming through DCs. - Induction of IL-12 and IL-15 production by DCs, responsible for clonal expansion and differentiation of CTLs - Facilitate shaping of CD8+ T cells into memory CTLs	- Differentiation into antigen-specific effector T cells is activated by DCs - Blockade of CTLA-4 and PD-1 can potentiate the activity of CD4+ T cells	- TGF-β suppresses proliferation	(26, 27)
Tregs	- Weakening the translocation of CTLs to the tumor nucleus - TGF-β release and suppression of CTLs activity - Expression of CD73 on the surface contributes to Treg-mediated inhibition of CTLs immunosuppressive activity - CTLA-4-mediated suppression of antigen-presenting cells - Consumption of IL-2 - Release of anti-inflammatory cytokines (IL-10, TGFβ)	- IL-6 - Gradients of the chemokines CCR4-CCL17/22, CCR8-CCL1, CCR10-CCL28, and CXCR3-CCL9/10/11 are recruited to the TME -TGFβ for the development, function, and survival of Tregs	- The PI3K-AKT-mTOR signaling pathway blocks the generation of Foxp3 + Tregs	(28, 29)
DCs	- Activation of CTLs through antigen cross-presentation - Transmission of costimulatory signals from CD4+ T cells to CTLs - Interaction with NK and B cells - Infiltration of DCs attracts immune effector cells	- CD4+ T cells can promote the activation and maturation of DCs - GM-CSF for recruitment, maturation and survival	- Activated by CCL2, CXCL1, and CXCL5 and VEGF released by cancer cells - Tumors can paralyze DCs through the induction of PD-1 expression - CTLA-4 is able to competently bind to CD80 and CD86 on DCs, preventing the activation of CD8+ T cells by DCs	(26, 27)
NK cells	-Immunosurveillance of tumor -Immature NK cells have antitumor activities -Differentiated NK cells with PD‐1 receptor exhibit protumor activities -Destruction of cancer cells exhibiting MHC-I expressive profile -Release of perforin and granzymes that induce cancer cell apoptosis -Secretion of pro-inflammatory cytokines and chemokines (GM-CSF, CCL5, TNF, IFN-γ, IL-6)	- IL-2 promotes tumor-led engagement of chemokines secreted by DCs (IL-12, etc.) - IL-2 promotes proliferation	- Reduction of NK cell infiltration by TGF-β and other immunosuppressive agents released by cancer cells - Limitation of function through inhibitory checkpoints	(27)
M1-polarized macrophages	- Antitumor action - Production of pro-inflammatory cytokines, reactive oxygen and nitrogen species	-IFN-γ, bacterial lipopolysaccharide (LPS), TNFα	- Blocking Notch signaling leads to M2 polarization	(27, 30)
M2-polarized macrophages	- Production of anti-inflammatory cytokines - Suppression of immunosurveillance against tumor cells - Promote angiogenesis and matrix remodeling, inducing tumor progression - Tumor-associated macrophages are considered a major source of MDSCs	-IL4, IL13, IL10	- SOCS3 (a downstream molecule of Notch signaling) promotes M1-polarization of macrophages	(27, 30)
Neutrophils	- Like macrophages, they have N1 and N2 polarization and contribute to anti-tumor and pro-tumor action, respectively - N1 neutrophils release granules with cytotoxic compounds to destroy cancer cells, secrete cytokines and chemokines. - N2 neutrophils are similar to polymorphonuclear MDSCs	- IL8 promotes neutrophil recruitment through binding to CXCR1 and CXCR2 receptors - DAMPs (HMGB, S100) overexpressed in tumors enhance neutrophil chemotaxis - GM-CSF, G-CSF and IFNg block neutrophil apoptosis	- TGFβ prevents the infiltration of N1-neutrophils into the tumor and promotes the accumulation of N2-neutrophils in the tumor	(31)
MDSCs	- Enhancement of angiogenesis through the production of MMP9, prokineticin 2 and VEGF - Induction of cancer cell migration to endothelial cells, metastasis - Inhibition of T-cell function through production of arginase, inducible nitric oxide synthase, TGF-β and IL-10 - Production of indole amine 2,3 dioxygenase (IDO), which suppresses immune response and induces Treg production	- GM-CSF, SCF-1, PGE2, COX-2, VEGEF, M-CSF, and IL-6 induce the expansion of MDSCs	- IDO inhibitors (1-methyl-L-tryptophan or STAT3 antagonist JSI-124) block MDSC immunosuppressive activity	(32)
B cells	- Production of cytokines that activate CTLs - Act as antigen-presenting cells - Production of cytokines that recruit MDSCs and enhance angiogenesis - Suppression of T-cells through IL-10 production	- Recognition of antigens via B cell receptors (BCRs) - Costimulatory molecules (CD80/86) - GTF-β can become immunosuppressive under the influence of TGF-β	- Polymorphonuclear MDSCs are able to suppress B-cell proliferation	(33, 34)

Tumor cells and tumor microenvironment use various strategies for immune evasion: disruption of antigen expression or presentation, accumulation of metabolites suppressing the functioning of effector T cells, modulation of cellular extracellular matrix to impede immune cell penetration and motility, the exposure of inhibitory immune checkpoints, secretion of immunosuppressive cytokines and production of chemokines recruiting pro-tumor stromal cells (35).

Depending on the composition of immune infiltrates and the nature of the inflammatory response, the TIME can be subdivided into three main classes: infiltrated-excluded (I-E), infiltrated-inflamed (I-I), and deserted TIME (36). I-E TIME is characterized by the presence of a variety of immune cells; however, due to the absence of specific cytokines and the presence of inhibitory molecules, effector immune cells — particularly cytotoxic T lymphocytes (CTLs) — are predominantly restricted to the tumor border (36). These tumors typically exhibit low expression of immune activation markers, such as granzyme B (GZMB) and IFN-γ in CTLs. In contrast, I-I TIME is densely populated with T cells, myeloid cells, and monocytes within both the tumor core and stroma, and is considered immunologically “hot.” This class is marked by high expression of PD-1 and CTLA-4 on T cells, PD-L1 on tumor cells, and elevated levels of pro-inflammatory cytokines (37). A notable subclass of I-I TIME is TLS-TIME, distinguished by the presence of tertiary lymphoid structures (TLS) that resemble lymph nodes in cellular composition, containing various lymphocytes (T cells, B cells, and dendritic cells). Conversely, the deserted TIME is considered immunologically “cold” or weakly immunogenic, due to the paucity of immune cells and cytokines both within the tumor core and at its periphery. The grade of TIME depends on the tumor’s characteristics and largely determines its immunological status and response to therapy. Notably, oncolytic viruses (OVs) have been shown to modulate the TIME, transforming tumors from an immunologically “cold” to a “hot” status (37).

## 4 Reinforcement of cell death with OVs

Oncolytic viruses can induce immunogenic cell death (ICD) in tumor cells, but their efficacy can be enhanced through genetic modification and their combination with other therapies. These strategies can potentiate their immunostimulatory effects at different stages of the cancer-immunity cycle. Here, we describe the main types of genetic modifications that stimulate ICD and enhance the immune response.

### 4.1 OVs engineering for ICD modulation

Different oncolytic viruses (OVs) possess unique proteins that modulate cell death; however, there are common patterns in their mechanisms of action, which depend on the type of cell death induced and the associated effector signaling pathways. Several strategies have been used to modify OVs for the promotion of ICD, including the deletion or knockout of genes, the insertion of genes that modulate cell death, and the arming of OVs with immunostimulatory molecules, such as TAAs, DAMPs, co-stimulatory ligands, cytokines, and chemokines. Numerous reviews have discussed the various types of cell death and their modulation by viruses (18). Viruses are well known for their intrinsic ability to influence cell death pathways; conversely, activation of specific cell death pathways can significantly impact the viral life cycle. For example, downregulation of autophagy-related genes (ATG5 and ATG10) reduces the ability of adenovirus to induce cell lysis (19). Engineering gene-deleted variants of adenovirus may enhance oncolytic potential compared to wild-type viruses. Examples of wild-type, attenuated, and recombinant OVs that activate various forms of ICD are presented in **Table 2**.

**Table 2:** Types of ICD induced by oncolytic viruses.

ICD type	Virus	Modification	Results	Ref.
Apoptosis	Adenovirus (AdΔE1B19K)	Deletion of *E1B19K* gene	Enhancement of gemcitabine-induced apoptosis in the pancreatic carcinoma cells PT45 and Suit2 and in PT45 xenografts	(38)
	Herpesvirus (vBSΔ27)	Deletion of *ICP27* gene	Induction of apoptosis in human cells	(39)
	Vesicular stomatitis virus (VSV-ΔM51)	Deletion of methionine at amino acid position 51 of the M protein	Induction and enhancement of apoptosis through type II extrinsic pathway in pancreatic ductal adenocarcinoma cell lines	(40)
	Vaccinia Virus (VG9-IL-24)	Disruption of the viral thymidine kinase gene region, IL-24 expression	Induction of apoptosis in breast cancer cell lines through PI3K/β-catenin signaling pathway. Delayed tumor growth and improved survival in MDA-MB-231 tumor model	(41)
	Newcastle disease virus (rAF-IL12)	IL12 expression	Induction apoptosis of CT26 colon cancer cells, cell cycle arrest at G1 phase. CT26 tumor growth inhibition in Balb/c mice, increasing the level of CD4 + , CD8 + , IL-2, IL-12, and IFN-γ.	(42)
	Measles Virus (rMV-BNiP3)	*BNiP3*, human pro-apoptotic gene, insertion	Induction of apoptosis in breast cancer cells (MDA-MB-231, MCF-7)	(43)
Autophagy	Adenovirus (Ad-hTERT-E1a-apoptin; Ad-VT)	*E1A* gene is driven by the cancer-specific promoter hTERT, apoptin expression	Regulation of autophagy through the AMPK-mTOR-eIF4F signaling axis. Reduction of drug resistance of MCF-7/ADR adriamycin resistant human breast cancer cell line). Decreased tumor volume in BALB/c with SGC7901 cell line (human gastric cancer), increased survival of mice	(44, 45)
	Measles Virus (rMV-Hu191)	Attenuated measles vaccine strain	Promotion of caspase-dependent apoptosis and complete autophagy through PI3K/AKT pathway in human colorectal cancer cells	(46)
Necroptosis	Adenovirus (ZD55-IFN-β)	IFN-β expression	Initiation of caspase-dependent apoptosis and necroptosis in human hepatoma cells SMMC-7721	(47)
	Adenovirus (dl922-947)	E1A CR2 deletion	Induction of necrosis in cancer cell lines	(48)
Ferroptosis	Newcastle disease virus	Wild type	Induction of ferroptosis through p53-SLC7A11-GPX4 pathway in U251 cells	(49)

### 4.2 TAAs and DAMPs

Classical apoptosis and autophagy are generally considered as non-immunogenic cell death; hence no DAMP, PAMP, TAAs and TSAa are released. OVs induced ICD of cancer cells stimulate the release of immunogenic molecules that are recognized by APCs, which subsequently activate cytotoxic T-lymphocyte (CTL) response. To stimulate a specific immune response, OVs are used *in situ* as vectors of TAAs. For example, adenovirus expressing human dopachrome tautomerase (hDCT) leads to a potent CTL response in a mouse model of melanoma (50). In another study, oncolytic vaccinia virus carrying the immunodominant major histocompatibility complex class I restricted H-Y antigen epitope also stimulated a systemic CTL response in the MB49 murine model of bladder cancer (12). However, since many TAAs are also present in healthy tissues, there is a risk of autoimmunity (51). Therefore, it is necessary to localize the action of OVs in the tumor.

DAMPs similarly to TAAs trigger an immune response in tumor microenvironment through recognition by APCs, which allows them to be used to reinforce OVs to boost antitumor immunity. For example, Measles virus (MV) encoding *Helicobacter pylori* heat shock protein A (HspA) has been shown to have enhanced replicative activity and exhibit a pronounced antitumor effect in *in vitro* and *in vivo* models of ovarian cancer (52). Recombinant adenovirus AdSurp-Hsp70 encoding the Hsp70 gene under the regulation of Survivin promoter demonstrated selective replication and lysis of survivin-positive gastric cancer cells and inhibition of tumor growth inhibited tumor growth of gastric cancer xenografts in immunodeficient and immune-reconstructed mouse models (53).

### 4.3 Cytokines and chemokines

Cytokines, including chemokines, play a critical role in the cancer immunity cycle, in which they act as signal transmitters between cellular components of TIME and contribute to both induction and suppression of immunity (**Table 2**). Cytokines have been widely utilized in tumor virotherapy and are being actively studied (54). Perhaps the best-known example is Talimogene laherparepvec (T-VEC), an attenuated herpes simplex virus, type 1 (HSV-1) expressing human granulocyte-macrophage colony-stimulating factor (GM-CSF), approved by the FDA for melanoma treatment. GM-CSF has also been actively used to reinforce various OVs (55). However, modulation of TIME by GM-CSF remains ambiguous: while GM-CSF stimulates DCs maturation and M1-polarized macrophage activity, it may promote tumor progression (56). Various immune-activating cytokines and chemokines (i.e. CXCL11, CCL2/5/19, FLTL3, IFN-α/β/γ, IL2/7/12/15/18/23/24, TNF-α, etc.) are being investigated on preclinical tumor models (neuroblastoma, breast tumor, lymphoma, lung cancer, glioma, melanoma, etc.) and in clinical trials (35).

### 4.4 Co-stimulatory ligands

Another method to enhance the antitumor immune response is to activate immune cells through the expression of co-stimulatory ligands by OVs (e.g., CD40L, 4-1BB, CD80, ICOS ligand) or, conversely, inhibitors or antagonists of co-inhibitory molecules (e.g., antibodies against “don’t eat me” signals (CD24 and CD47) used by the tumor for immune evasion) (57).

### 4.5 Combination with other therapies

The tumor’s significant defense system against OVs, including restricted viral spread, resistance to oncogenic signaling pathway targeting, and immunosuppressive tumor microenvironment, often limits the effectiveness of OV monotherapy (58). To enhance therapeutic efficacy, oncolytic virotherapy is frequently combined with other treatment modalities, including conventional therapies, such as external beam radiotherapy, targeted radionuclide therapy, and chemotherapy; biological therapies utilizing small molecules that modulate the innate antiviral response or cell death pathways; and immunotherapies, notably antibody-mediated immune checkpoint blockade targeting CTLA-4, PD-1, and PD-L1 (59). Among these, the combination of oncolytic viruses with immune checkpoint inhibitors has shown particular promise, with clinical trials, demonstrating improved objective response rates and the potential to convert immunologically “cold” tumors into “hot” ones, thereby increasing sensitivity to immunotherapy (11).

One of the major challenges in virotherapy is the induction of immune responses directed primarily against viral antigens rather than tumor antigens, which can limit the antitumor effect. A potential solution is the “prime-boost” strategy, in which an initial dose of virus encoding a tumor antigen primes the immune system, and subsequent doses further amplify, or boost, the antitumor immune response (58).

## 5 Targeting technologies for arming oncolytic viruses

There is no doubt that viruses are able to bind both normal “healthy” and malignant cells. However, oncolytic viruses productively replicate specifically in cancer cells while their productive infection is hampered in normal cells. Virus tropism is determined by cell receptors and/or co-receptors that viruses use to initiate cell attachment followed by cell entry. For example, the measles virus has a natural tropism for the human CD46 molecule, which facilitates virus binding to the cell (60). Despite ubiquitous expression of CD46 in all nucleated cells, its overexpression in cancer cells increases tumor susceptibility to the measles virus (61). Nevertheless, many receptors used by viruses are also expressed on non-malignant cells, allowing the possibility of infection. Therefore, an important focus of OV modifications and targeting technologies should be ensuring the specificity of OV against cancer cells.

**Figure 2:**
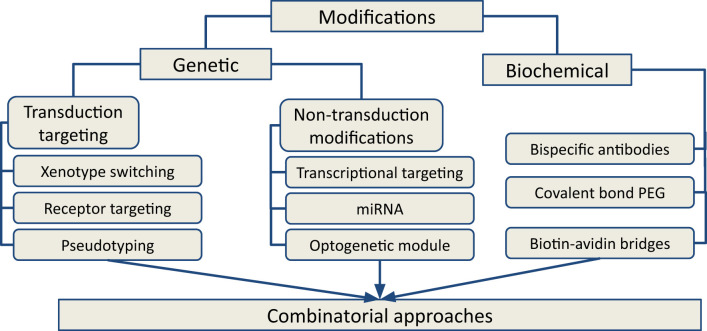
Types of approaches for OVs modifications for next-generation cancer therapy. Genetic and biochemical modifications are two major categories that grouped the available approaches. Based on the mechanism of action, the approaches could be clustered in transductional targeting and non-transduction targeting groups. Combinatorial approaches using various modifications of OV occupy a central place in current oncolytic therapy.

**Figure 3:**
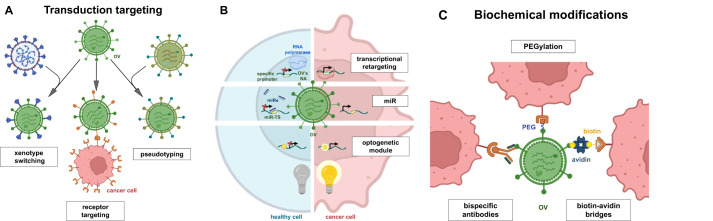
Modifications of OVs by genetic and biochemical methods. Genetic modifications can be divided into two groups: transduction targeting **(A)** and non-transductional targeting **(B)**. Transduction targeting may use xenotype switching, cell receptor targeting and virus pseudotyping. Non-transduction targeting approaches focus on transcriptional retargeting, RNA interference (miRNA) or control of gene expression using optogenetic technologies. Variety of biomedical modifications **(C)** have been applied to increase OVs specificity (e.g. ligand conjugation, bispecific antibodies) to cancer cells and increase its potency (PEG, biotin-avidin bridges).

Available approaches for OV modifications in research and development are broadly divided into two categories: genetic and biochemical targeting (**Figure 2**). Genetic modifications are more challenging to design and implement but, unlike biochemical modifications, they ensure that the desired features are reproduced in the viral progeny. Genetic modifications are further classified into transduction-targeting and non-transduction targeting approaches (**Figure 3**). These changes are intended to preserve the virus’s oncolytic efficacy while preventing it from harming healthy cells. On the other hand, the virus can be modified to show very little tropism for the target cell type. This approach reduces the virus’s toxicity to healthy cells, but it requires a deep understanding of the host mechanism that restricts the spread of the virus and the availability of accessible targets in order to overcome these restrictions.

### 5.1 Transduction targeting

Transductional targeting has been studied since the 2000s and involves various methods for modifying oncolytic viruses to maximize their transduction of tumor cells. The key goal for transduction targeting is to minimize OVs’ replication in non-cancerous cells. Therefore, thorough screening of viral interactions with healthy cells is essential. The genetic approach includes modifying viral capsid proteins to specifically target tumor cells while reducing entry into non-tumor cells. This is accomplished by genetically altering existing viral proteins and inserting new genes that encode interaction proteins, which enhance the OVs ability to bind to the surface of cancer cells. In the following sections, we provide summarized transduction targeting approaches proposed for OV therapy.

#### 5.1.1 Genetic targeting of OV on tumor cell receptors

One branch of transduction targeting involves interactions with tumor surface markers, such as the avβ6 integrin, which is expressed in a number of aggressively transformed epithelial cancers but remains undetectable in healthy tissues (62, 63). This approach has been used to modify adenoviruses (Ad or HAdV). Vectors based on serotype 5 (HAdV-C5, Ad5) are the most commonly used. According to Bates et al. the tumor tropism of Ad5 on avβ6 was achieved by genetic insertion of a 20 amino acid peptide, A20 (NAVPNLRRGDLQVLAQKVART), which is native to foot-and-mouth disease virus, into the fiber knob DG protein of Ad5 adenovirus (62). Modified A20 viruses selectively infect cells expressing integrin avβ6 - epithelial carcinoma cells, including ovarian, breast, pancreatic and colorectal cancers (64–66). The avβ6 integrin stimulates tumor metastasis and invasion through TGF-β activation and is associated with poor prognosis (67). Therefore, targeting avβ6 integrin may be beneficial for reducing metastasis. Wild-type adenovirus 5 is characterized by poor tumor selectivity, premature sequestration, and immune inactivation, which reduces the clinical efficacy of HAdV-C5. Specifically, HAdV-C5 binds to the adenovirus CAR receptor, ubiquitously expressed in healthy cells (68), entering via avβ3/5-mediated integrin internalization (69). Additionally, HAdV-C5 interacts with clotting factor X (FX) in the blood via the hexon protein, mediating virus transduction into hepatocytes and leads to hepatotoxicity (70). These unwanted interactions were eliminated by introducing a mutation in each major capsid protein. For instance, a mutation in a fiber called KO1 is known to eliminate CAR binding (71), while RGD > RGE mutation in the penton base prevents binding to avβ3/5 integrins and the hexon responsible for FX binding was modified to prevent interaction with FX (70). In addition to targeting the tumor-associated integrin, these genetic modifications allowed efficient and selective transduction of avβ6+ and epithelial ovarian cancer cell line (EOC). It was confirmed in a human SKOV3 EOC peritoneal xenograft model in mice. Animals receiving the novel oncolytic Ad5NULL-A20 showed significantly better survival compared to animals treated with oncolytic Ad5 without the A20 insert (63). In most populations, the high seroprevalence of Ad5 significantly reduces the efficacy of oncolytics based on it (72). Therefore, Bates et al. (62) applied the same modifications for Ad5NULL-A20 to the rarer serotype HAdV-D10 isolated from the ocular mucosa of patients with conjunctivitis (73), which has a seroprevalence rate of about 10% in a European cohort. Because HAdV-D10 naturally has a very low tropism to CAR, does not utilize DSG2 as a cellular receptor, and has a low, insufficient affinity for CD46 for cell penetration, residual off-target interactions with CAR on platelets and erythrocytes are readily eliminated by KO1 modification (74), and replacement of an RGD > RGE further reduces uptake in the spleen (75). Intratumoral administration of such mutant adenoviruses (HAdV-D10.A20) to mice bearing BT20 xenografts (expressing avβ6 integrin) showed a significant reduction in tumor volume 9 days after administration, compared to placebo and wild-type HAdV-D10 serotype (62). Another example of an oncolytic virus targeting tumor markers involves the targeting of herpes simplex virus (HSV) to the epidermal growth factor receptor (EGFR). Apolloni et al. (76) engineered R-613, the first oncolytic HSV to specifically target EGFRvIII (a variant of the epidermal growth factor receptor commonly mutated in glioblastoma). Additionally, another genetically modified OV, OV-Cmab-CCL5, expresses a single-chain variant antibody fragment specific to EGFR. OV-Cmab-CCL5 is redirected to EGFR - positive glioblastoma (GBM) cells and facilitates continuous production of the cytokine CCL5 in the tumor microenvironment. In addition to efficient targeting, infection of GBM cells with OV-Cmab-CCL5 significantly enhances the migration and activation of natural killer cells, macrophages, and T cells. Furthermore, it suppresses tumor-specific EGFR signaling, reduces tumor size, and prolongs the survival of GBM carrier mice (77). A series of studies focused on retargeting the tropism of herpes simplex virus (HSV) types 1 and 2 to the human epidermal growth factor receptor 2 (HER-2) oncoprotein (78). For instance, Nanni et al. (79) targeted HSV1 to HER-2p185, which is overexpressed in ovarian and breast cancers. In the virus genome, they replaced part of the sequences encoding the receptor-binding glycoprotein gD (a major factor in HSV entry into healthy cells) with antibody fragments targeting the HER-2 oncoprotein overexpressed in human breast and ovarian cancer, thus completely eliminating the natural tropism of HSV. The resulting R-LM249 virus infects and kills exclusively tumor cells that express high levels of human HER-2. Intraperitoneal administration of R-LM249 to immunodeficient mice (Rag2-/- and Il2rg-/-) with implanted human SK-OV-3 ovarian carcinoma cells (a model that mimics the fatal condition in patients with advanced stages of the disease) significantly inhibited carcinomatosis. Sixty percent of the treated mice showed no peritoneal diffusion and the total weight of neoplastic nodules was reduced by 95%. Intraperitoneal administration of R-LM249 also suppressed the growth of HER-2+ breast cancer MDA-MB-453 cells that had metastasized to the brain and ovaries. The authors considered these findings promising for tumor therapy, although they require confirmation in clinical trials (79). The urokinase-type plasminogen activator receptor has also recently attracted interest as a target for oncolytic viruses in pancreatic cancer (80).

#### 5.1.2 Transduction targeting by chimeric and pseudotyped OVs

A significant number of studies have focused on the construction of viral chimeras (hybrids) - the combination of viral particles and proteins of different viruses to improve the properties of oncolytic viruses (OVs) (81). Chimerism can include any transgenic viruses with combined properties, including “fiber mosaicism” (82), pseudotyping - exchange of envelope glycoproteins or whole capsids (83) (**Figure 3**) and/or xenotype switching (interspecies combinations of viral capsid components) (84). Vesicular stomatitis virus (VSV) is a good example of an animal virus that has been successfully engineered for safe therapeutic applications in oncology. VSV is capable of infecting a broad spectrum of cell types and demonstrates rapid replication kinetics, which enhances its capacity to disseminate within tumors and induce robust oncolysis. Importantly, VSV is non-pathogenic to humans and exhibits preferential replication in malignant cells due to the impairment of type I interferon (IFN) signaling pathways in cancer cells (85, 86).

One of the most popular trends is the creation of adenovirus hybrid fibers. Adenoviruses attach to the target cell via the knob domain of the trimeric capsid protein fiber. This domain is structurally conserved across different Ads serotypes but utilizes several different receptors. The interaction of Ads with the receptor depends on both fiber length and flexibility (87). For example, fiber length influenced the bio distribution of Ads in mouse models of ovarian cancer, increasing the tumor-to-liver transduction ratio about 10-fold when HAdV-5-based virus was used with a shorter fiber from HAdV-3 to HAdV-5/3 (88). Replacing the HAdV-5 fiber with the HAdV-3 fiber changes not only the tropism to cellular receptors, but also significantly improves binding and cell penetration (89). For example, transduction levels of HAdV-5/3 primary melanoma cells increased by three orders of magnitude compared to HAdV-5 controls (90). Similarly HAdV-5/16 and HAdV-5/50 chimeras show specific tropism to pancreatic cancer cells (91). When combined with transcription control elements, these fiber chimeric viruses exhibit high specificity to target cells, such as the Ad targeting melanoma shown by Rivera et al. (92). Besides HAdV-5 chimeras, HAdV-35 chimeras with other serotypes are popular (93). It is also possible to replace the fiber entirely, provided that it can be incorporated into the capsid. For example, the TRAIL-armed (tumor necrosis factor-related apoptosis-inducing ligand) oncolytic virus HAdV-5/35 induced superior tumor shrinkage after both systemic and intraperitoneal administration in *in vivo* models of cervical cancer (94). An Ad5LacZ vector, incorporating a mutation in the hexon variable region (HVR7) and pseudo typed with the Ad35 Ad5CMV-HCR5*7*E451Q/F35++ fiber (termed Ad5T*F35++), devoid of FX binding and redirected from CAR to CD46, demonstrated significantly higher transduction of EOC cells compared to the parental Ad5 vector (95). Fiber chimerism can be used to modify transduction specificity in a more indirect way. For example, HAdV-41 has features of both long and short fiber phenotypes. Cell binding and transduction depend exclusively on the long fiber. Ads with short fibers cannot bind to receptors and are eliminated *in vitro* and *in vivo*. If HAdV-5 is modified with short fiber HAdV-41 (HAdV-5/41s), it will lead to disadaptation of such viruses (96). As a result, “blind” adenoviruses can function as a framework for retargeting novel receptors (97). The capsid components of viruses can be replaced not only by equivalent domains of species-specific serotypes, but also by components of viruses of other species. The strategy of obtaining interspecific hybrids has been termed “xenotype switching”. Thus, the transduction efficiency and tropism of adenoviruses *in vivo* were improved by using knob domains from Ad dogs - CAdV-2 (84), or by fiber exchange with Ad sheep - OAdV-7 (84). A HAdV-5-based vector with a chimeric fiber incorporating a HAdV-2 site and a bovine BAdV-4 (Ad5FB4) fiber is of interest, as this fiber binds to membrane proteins of the B7 family overexpressed by murine leukemia cells derived from dormant tumors (98). The possibility of modulating tropism by creating chimeras has also been considered in other viral systems. Pseudotyping of adeno-associated viruses (AAV) to expand their tropism typically involves incorporating a vector genome derived from AAV2 into the capsid of another serotype, such as AAV5 (99). This combination allows for enhanced transduction during gene transfer into the lungs (100). Additionally, Tyrosine residues (101) and other kinase targets such as serine, threonine, and lysine on the AAV capsid can enhance *in vitro* transduction efficiency and also lead to improved transgene expression (102). Incorporating designed ankyrin repeat proteins (DARPins) (103) or a nanobody (104) into the AAV capsid has enhanced specific targeting of B cells (103). The pseudotyping strategy has also been applied to retroviral and lentiviral vectors, which show low transduction efficiency. Increased specificity can be achieved by exchanging glycoproteins with other enveloped viruses. The first of such a chimera was Moloney Murine Leukemia Virus, pseudotyped with the glycoprotein of vesicular stomatitis virus (VSV-G) (83), characterized by high transduction efficiency and broad tropism. Other glycoproteins for pseudotyping have origins in Sindbis virus (105), paramyxovirus (106), rabies virus (107), and measles virus (MV) (108). Pseudotyping with MV-glycoproteins opens the possibility of antibody-mediated retargeting and enables specific transduction of both antigen-expressing target cells in mixed cell cultures and resting primary lymphocytes (109). In addition, MV-glycoproteins have been utilized to repurpose VSV for virotherapy, in which broad VSV-G-mediated tropism is disadvantageous (110).

#### 5.1.3 Biochemical retargeting of OVs

Another approach to achieving virus specificity is biochemical retargeting, which involves conjugation with cell-targeting ligands. This includes, for example, the use of bispecific antibodies, biotin-avidin molecular bridges, and pegylation. This approach stands out because it does not lead to complications associated with genetic modifications and allows targeting the virus to several cell receptors simultaneously. The main disadvantage of the method is that the modification is limited to one generation of the virus and is not transmitted to the offspring after replication (111). One example of such modification, Ad5 targets the EGFR, which is overexpressed in many types of head and neck squamous cell carcinoma (HNSCC) (112). Upon binding to one of its seven native ligands, EGFR is activated, dimerises and promotes cell proliferation, contributing to tumor growth (113). To achieve specific targeting of EGFR by human adenovirus type 5 (HAdV-5)-based vectors, the affinity ligand EGFR affilin was covalently attached at a specific position to either the fiber knob or the hexose capsid protein. *In vitro* and *in vivo* studies investigated the transduction of EGFR-specific cancer cells as well as susceptibility to natural sequestration mechanisms, pharmacokinetics and biodistribution profiles of affilin-decorated vectors (114). *In vitro* studies provided results supporting the concept that covalent attachment of a receptor-specific affilin to the adenoviral capsid provides an efficient and versatile tool for adenoviral vectors to target cancer-specific receptors. However, in the case of EGFR as a vector target, transduction into non-target tissues and the low availability of the receptor in tumor tissues prevented effective tumor transduction by affilin-decorated vectors, making EGFR a difficult receptor to target with adenoviral vectors (114). A similar approach to biochemical targeting is the modification of tumor ligands to facilitate interaction with OVs. One of such modifications is sialylation, the enrichment of tumor surface glycans with sialic acid residues. In a study on breast cancer (115) using nanobiophysical approaches, it was shown that overexpression of α-sialylated glycans, by altering their mechanical properties, enabled reovirus attachment and infection in a serotype-dependent manner. Co-injection of α-sialylated glycans and reovirus increased reovirus infectivity in malignant cells. This study confirmed both the use of reoviruses as oncolytic agents in nanomedicine and the role of α-sialylated glycans as adjuvants in enhancing oncolysis, opening new perspectives in oncolytic cancer therapy (115). Recent reviews discuss the topic of cell modification in great detail, particularly in relation to enhancing the efficacy of oncolytic viruses (116).

### 5.2 Non-transduction targeting

Non-transduction targeting is the modification of the virus genome to enable replication exclusively in cancer cells. The strategy includes approaches, such as transcriptional targeting, targeting matrix metalloproteinases (MMP) and targeting miRNAs.

#### 5.2.1 Transcriptional targeting

Transcriptional targeting is most often achieved by placing critical parts of the viral genome under the control of tumor-specific promoters, which are active in tumors but inactive in most normal tissues. Cancer-specific promoters are primarily applicable to engineer DNA-genome OVs (e.g. herpesviruses, adenoviruses). For example, the human epidermal growth factor receptor 2 (HER-2) promoter is often used to regulate the replication of herpes simplex virus (HSV) in glioma cells (79). For HSV-based oncolytics, the detargeting/retargeting strategies used so far are based on genetic modifications of glycoprotein D (gD), where specific amino acid residues (30, 40) are deleted and amino acid 38 is replaced with a single-chain fragment of the scFv antibody to the cancer marker HER-2 (117). HER-1 is also highly suitable for regulation by the surviving promoter (118). Transcriptional control of the viral alpha 4 gene encoding infected cell protein-4 (ICP4) by the Survivin/BIRC5 cellular promoter conferred a tumor cell-restricted replicative potential to the virulent genome of HSV-1 (118). Sasso et al. generated a dual-regulated HSV-1 in which tumor cell-restricted replicative potential was combined with selective transduction via ERBB2 HER-2 receptor retargeting (119). In addition to targeting replication in cancer cells, detargeting the tropism of HSV from natural herpesvirus entry mediator (HVEM) and nectin receptors is used to prevent off-target and extra-tumor infections (120). Combination strategies, including transcriptional retargeting/detargeting and post-transcriptional retargeting, have proven to be highly effective in achieving tumor specificity while preserving virulence of the virus (121). Expression of HSV proteins (such as γ34.5,ICP4, ICP27 and UL8) outside the tumor can be prevented by inserting targeted miRNA sequences or tissue-specific promoters into the virus genome (122). For example, due to the hybrid nestin enhancer-HSP68 minimal promoter, expression of the virulence gene γ34.5 was restricted to nestin-positive glioblastoma cells (123).

The cell mass of solid tumors develops predominantly in a hypoxic environment, therefore, a number of studies (as shown in **Table 3**) have focused on regulating the expression of the toxic adenovirus E1A protein through regulatory elements responsive to hypoxia. The E1A gene was placed under the control of various tissue-specific tumor promoters for controlled cytotoxic effects (**Table 3**). One of such promoters is the human telomerase promoter (human telomerase reverse transcriptase hTERT, see **Table 3**), as telomerase activity is often higher in cancer cells than in normal cells (133). Another example is the replacement of the E1A promoter with the promoter/enhancer of the melanocyte- and melanoma-specific tyrosinase gene (137). At the same time, mutations were introduced into the E1A gene itself that prevented interaction with the retinoblastoma protein pRb and the coactivating protein p300, thus excluding virus replication in normal cells (AdTyrΔΔ2Δ24). As a result, AdTyrΔΔ2Δ24 showed Ad-specific replication, and killed *in vitro* exclusively melanoma cells. AdTyrΔΔ2Δ24 was further enhanced by incorporating a tyrosinase promoter to trigger the E4 gene (Ad2Xtyr), and the efficacy of this approach was demonstrated in a number of organotypic *in vitro* cultures (137). More examples of promoters used for tumor-selective replication of Ads and their expression of E1A are listed in **Table 3**.

**Table 3:** Promoters for cancer-specific adenovirus replication and E1A expression.

Promoter	Target tumor	Reference
MUC1	Breast cancer	(124)
Promoters responding to hypoxia and estrogen	Breast cancer	(125)
Prostate-specific antigen (PSA)	Prostate cancer	(126)
Probasin	Prostate cancer	(126)
α-feto-protein	Liver cancer	(127)
Regulatory sequence PPT (PSA enhancer, a PSMA enhancer and a T-cell receptor γ-chain)	Prostate cancer	(128)
Survivin	Malignant gliomas	(118, 121)
β-catenin-responsive promoters	Colorectal cancers Liver cancers	(129, 130)
Cyclooxygenase-2 (Cox-2)	Colorectal cancer Pancreatic cancer	(131, 132)
Human telomerase (hTERT)	Gastrointestinal cancer	(133–135)
PEG-3gene (PEG-Prom)	Primary and distant pancreatic tumors	(136)

#### 5.2.2 Targeting matrix metalloproteinases

Host cell proteases are determinants for the replication and pathogenicity of enveloped viruses (111). For example, host proteases activate the envelope protein ENV inHIV-1, the spike glycoprotein of SARS-CoV-2. The proteolytic cleavage of the envelope liberates the functional domains, allowing membrane fusion and entry into the cell (138). The proteases that are overexpressed by cancer cells can be targeted by oncolytic viruses, such as matrix metalloproteinases (MMPs). By attaching blocking ligands to MMP-cleavable linkers at the amino (N)-ends of retroviral glycoproteins, viral transduction was inhibited, providing evidence in favor of MMP activation. The common furin proteinases and/or tryptase from Clara airway secretory cells typically cleave the MV and Sendai virus fusion proteins specifically (139, 140). After reprogramming MV from these proteinases to MMP with minimal structural changes to the fusion protein, recombinant MV expressing the modified fusion protein (MV-MMP) did not multiply or exhibit cytotoxic effects in cells that did not express MMP. When injected into MMP-positive subcutaneous tumors in mice, MV-MMP maintained its complete oncolytic activity. After cerebral injection, MV-MMP did not infect or kill susceptible mice like wild-type MV did, indicating the increased safety of recombinant virus for the nervous system (141). Targeting oncolytic viruses to MMP-positive cancer cells at the level of viral particle activation may be safe and beneficial, as demonstrated by these experiments. Urokinase-type plasminogen activator is another protease that can be targeted in invasive metastatic cancer cells (142).

#### 5.2.3 MicroRNA targeting

MicroRNAs (miRNAs) are a class of short (∼22 nucleotides) non-coding RNA molecules that play a crucial role in epigenetic regulation (143). In normal cells, miRNAs regulate processes, such as cell differentiation, proliferation, apoptosis, and other processes. Changes in the expression profile of microRNAs have been found in the development and progression of most malignant neoplasms. They can act as both oncogenes and tumor growth suppressors (144). To date, numerous microRNAs have been identified with both increased and decreased expression in tumors of different locations. Individual microRNAs and their combinations have been proposed as diagnostic markers and prognostic factors, and their potential use as tumor growth inhibitors is being actively explored (145). The strategy for limiting viral pathogenicity to healthy tissues involves using miRNA from normal cells to restrict viral replication. This is done by interacting with miR-specific tissue sites (miRNA-TS) inserted into the viral genome. For example, members of the picornavirus family are promising candidates for cancer treatment due to their potent oncolytic activity. However, their replication is not limited to tumor cells and can occur in a variety of normal tissues. To enhance the safety of these OVs, miRNA-TS, which are expressed in normal tissues but are absent (or minimally present) in cancer cells, were inserted into the 3’ non-coding regions of the viral genome. In normal tissue, miRNAs inhibit viral activity via miRNA-TS in the viral genome by translationally repressing or catalytically degrading viral RNAs (146). This inhibition does not occur in cancer cells, where these miRNAs are absent or depressed at low levels.

Another example of miRNA targeting is the oncolytic vesicular stomatitis virus, which is highly tumor-specific due to its susceptibility to the interferon response in normal tissues. However, VSV is highly neurotoxic not only in rodents but also in non-human primates (147). Therefore, it is necessary to restrict VSV replication in the brain without altering the oncolytic effect of the virus. Kelly et al. (148) inserted a miRNA-125 targeting site into the L gene, which completely rescued cell viability in astrocytes. In contrast, inserting it into the M gene only resulted in a 30% rescue. Impressively, 90% of the mice infected with this recombinant virus survived without exhibiting any neurotoxic effects, and the virus’s oncolytic activity against other cancer cells remained unaffected (149). Additionally, another studydescribes how miRNA-TS can be easily inserted into the RNA of the Coxsackievirus B3 (CVB3) genome using fusion cloning technology (150). See more examples of miRNA and OVs in **Table 4**.

**Table 4:** MicroRNA (miRNA) for detargeting OVs from nonmalignant cells.

OV	miRNA	Reference
Adenoviruses	miRNA-122	(151–154)
Herpes Simplex Virus 1	miRNA-143 miRNA-145	(155)
Influenza A virus	miRNA-93	(156, 157)
VSV	miRNA let-7, miRNA 9, miRNA-26, miRNA-29, miRNA-125	(149, 158–160)
Picornavirus	miRNA-124, 125, miRNA-142	(161)
CVB3	miRNA-216, miRNA-375, miRNA-34, miRNA-1, miRNA-133	(150, 158, 159, 162–165)
CVA21	miRNA-142, miRNA-133, miRNA-206	(148, 166, 167)
Semliki Forest virus	miRNA-214	(168)
AAV	miRNA-1d, miRNA-206, miRNA-122	(169–172)
Measles virus (MV)	miRNA-122, miRNA-7, miRNA-148a	(173, 174)
Mengovirus, vMC24	miRNA-124, miRNA-133, miRNA-208	(175–178)
Lentiviral vectors	miRNA-155, miRNA-223	(179–181)
Poliovirus	miRNA-124	(182)

#### 5.2.4 OVs modifications by optogenetic modules

A relatively recent non-transduction targeting method has been found in the application of recombination of oncolytic viruses. The method is based on the regulation of gene expression by exposure of transduced cells to light waves of a certain spectrum. The genetic construct responsible for the mechanism for implementing this method is called the optogenetic module (OM). Optogenetic modules incorporated into recombinant oncolytic viruses can help improve tumor selectivity, reduce toxicity, and regulate the expression of immunomodulators, tumor suppressor genes, tumor-associated antigens, and microRNAs. Controlling expression with specific wavelengths of light may, in the future open up new, safe options for treating recurrent cancers with oncolytic viruses. Viral vectors have previously integrated various types of OM. In the study by Hagihara and colleagues, they developed a tumor-specific replication-competent adenovirus in which the expression of adenovirus early region genes 1A (E1A) and 1B (E1B), essential for viral replication, is driven by the human telomerase reverse transcriptase (hTERT) promoter element (183). The E1A and E1B genes were inserted downstream of the Gal4 upstream activator sequence (UASG). When exposed to blue light, the GAVPO complex, comprising the smallest light-oxygen-volt (LOV) protein, VVD, Gal4 (Gal4 residues 1–65), and the p65 activation domain — homodimerizes, allowing Gal4 to bind UASG and subsequently triggers the expression of E1A and E1B (183). The resulting photoactivatable oncolytic adenovirus (paOAd) was evaluated in cancer cell lines (A549, H1299, HepG2) and in a subcutaneous xenograft model (HepG2 or H1299 cells and Rag2/Il2rg double knockout mice). PaOAd demonstrated an oncolytic effect equal to that of the parent adenovirus. A pronounced cytopathic effect induced by paOAd was detected in TERT-positive human small intestinal organoids (SIO). Despite the positive results, the hTERT promoter is not specific for cancer cells, raising concerns about the safety of viral replication in non-cancerous cells (183).

Another group of researchers developed an alternative Opt/Cas-Ad system designed to regulate and enhance a tumor suppressor gene (184). Opt/Cas-Ad is based on a recombinant modified adenoviral vector (AdK7) carrying dCas9 accompanied by a Cry2-CIBN module (Cry2: Cryptochrome-2; CIBN: N-terminal fragment of calcium and integrin-binding protein 1, dCas9: catalytically inactive Cas9). Optically controlled Opt/Cas-Ad increased the expression level of Dkk-3 mRNA under blue light illumination and induced Dkk-3-mediated apoptosis in a PC3 cell xenograft tumor model. As a result, the growth of subcutaneous PC3 tumors in mice was effectively suppressed by oncolytic virus and spatiotemporal light gene expression. The authors suggest that the Opt/Cas-Ad system could improve the efficacy and safety of current virotherapy, as well as expand the therapeutic potential of OM for cancer treatment (184).

However, the prevalence of endogenous antibodies against adenoviruses makes their systemic administration difficult for cancer treatment (185), so the use of other potential oncolytic viruses are preferable. For example, in a different approach, the photo-dimerizing proteins pMag and nMag Magnet (186) were inserted into two flexible polymerase (L) domains of measles virus (MeV) or rabies virus (RABV), thereby reducing the catalytic activity of the polymerase. Blue light-induced heterodimerization of pMag and nMag proteins promotes the formation of the ribonucleoprotein complex (RNA-dependent RNA polymerase, nucleocapsid and cofactor proteins) and triggers viral replication. Recombinant MeV and RABV containing photocontrolled L protein and EGFP showed accelerated replication when activated by blue light. The antitumor potential of MeV with photocontrolled viral L-polymerase (rMeV-EGFP-LDMH) was demonstrated in a xenograft model (MDA-MB-468 cells and BALB/c nu/nu mice). Intratumoral injection of rMeV-EGFP-LDMH, followed by blue light illumination, showed significant suppression of tumor growth with 100% survival in the treated mice (186). Although the virus models discussed demonstrate promising results, a key limitation of these optogenetic modules is their reduced efficacy in clinical applications due to the poor tissue penetration of blue light. In contrast, optogenetic modules that utilize red-shifted light (e.g., REDMAP, iLight, PhyA-FHY1, PhyB-PIF, BphS) offer a more promising and viable alternative, as they can penetrate tissues more effectively (187). Genetic modifications and chemical approaches have been used to improve viral cellular entry through the cancer cell-specific receptors. These modifications are meant to add ligands to the virus for improving the binding to the receptors of the cancer cells, and to block the binding to normal cells. There are some challenges for both approaches, for example, the ligand incompatibility with the symmetrical icosahedral form of some viruses. Both strategies can be used in combination (111).

Prescribing oncolytic viruses (OVs) to patients might still be challenging due to delivery issues. Most OVs require direct injection into tumors (intratumoral delivery) to maximize effectiveness and minimize off-target effects. This method is feasible for accessible tumors like melanoma but is difficult for deep-seated or hard-to-reach tumors, requiring specialized skills and equipment. Intravenous delivery is being explored but faces issues, such as rapid clearance by the immune system and unpredictable dosing at the tumor site.

Pre-existing immunity is another hurdle that may limit OVs’ applications. Some patients may have pre-existing immunity to the virus being used, which can neutralize the OV before it reaches the tumor, reducing therapeutic efficacy.

## 6 Discussion

The improvement and development of novel, potent, and safe anti-tumor drugs remain a top priority in translational cancer research. Despite the availability of effective cancer therapies, certain tumors exhibit poor responsiveness to existing treatments and are associated with low rates of positive outcomes among patients (146). Addressing the high heterogeneity of tumors, as well as the unique genetic backgrounds of individual patients, is essential for the advancement of targeted anti-cancer therapies and the realization of personalized medicine as a potential cure.

Given the mechanisms of action of OVs, combining virotherapy with other cytotoxic anti-cancer approaches is considered a promising direction for future cancer treatment. Notably, the combination of OVs with immune checkpoint inhibitors, such as PD-1 or CTLA-4 inhibitors — or with CAR-T and CAR-NK cell therapies has demonstrated synergistic effects, enhancing the immune system’s capacity to recognize and attack tumors. Clinical trials have shown that these combinations can be both safe and effective, with some studies reporting improved objective response rates and durable anti-tumor responses compared to monotherapies (6, 116). Similarly, preclinical and early clinical data indicate that combining OVs with adoptive cell therapies, such as CAR-T or CAR-NK cells, can further remodel the tumor microenvironment and potentiate anti-tumor immunity. However, further research is needed to optimize treatment protocols and confirm long-term benefits across different tumor types (188).

Another key feature of OVs is their modular structure, which enables the *de novo* design of viral carriers to deliver effector molecules specifically to cancer cells. These molecules can be encoded genetically within the viral genome or attached via biochemical interactions, allowing OVs to introduce novel therapeutic modalities into the tumor microenvironment and stimulate local immune responses against cancer-specific antigens or malignant cells. Importantly, OVs generally do not interfere with other anti-cancer treatments, making them well-suited for combinatorial strategies that may yield synergistic effects and potentially increase the proportion of patients who respond positively to therapy. By shaping the anti-cancer immune response, OVs can also be used in “prime-boost” regimens in conjunction with chemotherapy.

However, it is important to note that OVs are not yet a sufficient alternative to currently approved therapies. OV-induced inflammation and anti-viral immunity can limit their distribution and long-term application. Recent advances in computational modeling and simulation have facilitated the prediction and optimization of OV design. For example, phenomenological models have been developed to study viral spread within tumors and normal tissues, taking into account the varying capacities of different cell types to support viral proliferation. These tools are instrumental in guiding the development of more potent and selective oncolytic viruses for clinical use (189). The probability distributions provided the susceptibility rate different cell types towards viral infection. The model was tested with VSV and the simulations were run to predict the relationship between virus replication, activation of IFN-mediated defense responses and cytotoxicity. The computational data verified by experimental observations in a variety of tumor models confirmed that the VSV D51-attenuated virus will eradicate IFN non-responsive tumors, whereas normal populations or IFN-responsive tumors will be largely resistant. In other words, virus cytotoxic efficacy towards tumors and the additional risk of damage to the nonmalignant cells could be predicted that may help to mitigate the risk of immunotherapy using OVs and improve therapeutic strategies. Virus dynamics are an important asset to simulate virus - cell interactions and choose the strategy for following experimental verifications. Growing evidence suggests that computational modeling approaches are valuable tools in the biomedical engineering pipeline, including virotherapy (190). Although that simulations of such kind of complex interactions may not provide a definitive answer, it may help to assess the risks and predict the virus kinetics in different cell populations. Oncotherapy using oncolytic viruses combined with potent anticancer drugs, armed with immunotherapeutic agents and accompanied by computational tools could be a unique and promising approach to develop novel strategies for previously untreatable cancers.

The approaches described above not only enhance the activity of OVs but also improve their safety. One promising strategy to mitigate health risks involves incorporating genetic circuits into the OVs’ backbone. These circuits enable precise regulation of the timing and magnitude of the virulence genes expression, as well as controlled delivery of therapeutic payloads, thereby balancing efficacy with biosafety (191). Some researchers have generated a chemogenetic switch of rapamycin-inducible ST7 RNA polymerase expression system (FRB/FKBP) in a vaccinia virus vector, Dox-inducible expression (TetR/TetO) and cumate-inducible expression (CymR/CuO). The FRB/FKBP system consists of a T7 RNA polymerase that is assembled to a rapamycin-induced dimerization system, consisting of FKBP-rapamycin binding domain (FRB) and FK506 binding protein (FKBP), so when rapamycin is introduced, FRB is recruited to FKBP that puts the C-terminus near the N-terminal T7 RNAP and the gene can be transcribed under the regulation of T7 promoter. The system was tested in different cancer cell lines demonstrating an increase in gene expression when rapamycin was added, and it also worked with its analogs without affecting the virus growth rate. In animals, the addition of the analogs of rapamycin increased the expression of the genes in tumors but decreased over time while maintaining the virus present.

The approaches described above not only enhance the activity of OVs but also improve their safety. One promising strategy to mitigate health risks involves incorporating genetic circuits into the OVs’ backbone. These circuits enable precise regulation of the timing and magnitude of the virulence genes expression, as well as controlled delivery of therapeutic payloads, thereby balancing efficacy with biosafety. However, studies have shown that pre-existing antiviral immunity to viruses, such as NDV and adenovirus can actually enhance the antitumor immune response by retargeting antibody-virus complexes and activating tumor-directed CD8+ T cells (192, 193).

Pre-existing antiviral immunity and severe immunodeficiency may worsen the adverse effects of OV therapy. Usually adverse effects of virotherapy are generally mild to moderate, but can vary depending on the virus used, route of administration, and patient factors. The most commonly reported side effects include flu-like symptoms, fatigue, and nausea (194).

In oncolytic virotherapy trials for advanced melanoma, the most common adverse effects were fatigue, chills, nausea, diarrhoea, headache, myalgia, gastrointestinal, musculoskeletal and connective tissue disorders (195). In non-melanoma skin cancer and cutaneous lymphoma clinical trials (196), the most common adverse events were fever, flu-like symptoms, fatigue, nausea and vomiting, cytopenias, injection site reactions, dizziness, arthralgia, mild hypoxemia and hypotension.

Other types of therapies, such as chemotherapy, radiotherapy and immunotherapy also present adverse effects. The most common adverse events in chemotherapy were nausea, vomiting, dry mouth, hair loss, fatigue, numbness in fingers or toes, confusion, depression, loss of appetite, chest pain, diarrhea, dyspnoea, and rash (197). The radiotherapy side effects are depression, fatigue, and depending on the site of radiation, it can cause oral mucositis, dysphagia, hyposalivation, pneumonitis, fibrosis phase, diarrhea, rectal bleeding, incontinence, nausea and vomiting (198). The ICI’s secondary effects can be cutaneous (maculopapular rash, pruritus, dermatitis), digestive (diarrhea, enteritis, abdominal pain, fever, blood/mucus in the stool, nausea and vomiting), liver damage, thyroid disorders, hypophysitis, neurotoxicity, cardiotoxicity, renal toxicity and respiratory toxicity (199).

According to the clinical trials data, most adverse effects from using OVs are manageable and tend to resolve with supportive care. Severe complications are rare but highlight the need for careful patient selection, monitoring, and adherence to safety protocols during OV therapy.

Despite the initiation of more than 200 clinical trials investigating various OVs, the regulatory framework governing their administration remains underdeveloped. Key challenges include the lack of standardized clinical endpoints, validated biomarkers, and appropriate control arms in clinical trials, which complicate regulatory approval and integration of OVs into standard cancer care. Regulatory agencies, such as the FDA and EMA have issued guidance documents specific to gene therapy and oncolytic virus products, but the unique biology of OVs, such as their delivery methods, pharmacokinetics, and immunologic effects poses additional hurdles for clinical trial design and product standardization (11).

Another significant challenge associated with resistance to oncolytic virotherapy involves tumor cell-dependent IFN signaling. IFN-mediated antiviral pathways inhibit viral spread and replication, thereby limiting the efficacy of oncolytic viruses. This mechanism of resistance has been well documented for many OVs, particularly RNA viruses (200). Tumor cells with intact or upregulated IFN signaling are able to mount robust antiviral responses, often through activation of the JAK/STAT pathway and induction of interferon-stimulated genes (ISGs), which restrict viral replication and oncolysis (201). Bhatt and colleagues also systematically analyzed other mechanisms that contribute significantly to the resistance mechanisms that affect the efficacy of oncolytic therapy. Among the known mechanisms, such as hypoxia-mediated inhibition, APOBEC-mediated resistance, viral entry barriers, epigenetic modifications, the authors have highlighted the importance of the stromal compartment in the development of resistance to oncolytic therapy (202). Interestingly, this study also found that some viruses (i.e. VSV, HSV and Ad) are more likely to induce resistance than others. Furthermore, in another study, Huff et al. showed that APOBEC3 dysregulation in cancer cells can worsen the efficacy of virotherapy (203). Given that APOBEC enzymes interact with mRNA molecules, and therefore RNA oncolytic viruses may face similar resistance challenges. A recent study by Dai et al. presented promising data suggesting that at least some oncolytic resistance can be overcome. Using a BET inhibitor and a mouse glioma model, they showed that HSV oncolytic activity is enhanced by neutrophil extracellular traps (NET) induced by IGF2BP3 (204). Increased NET formation in malignant tumors may have great therapeutic potential for virotherapy.

Taking together, advances in genetic engineering allow OVs to be modified for enhanced tumor specificity, improved immune activation, and delivery of therapeutic genes directly to cancer cells. Strategies include engineering viruses to express immunostimulatory molecules (such as chemokines and cytokines), modifying viral capsids for targeted delivery, and using nanomaterials as carriers to improve delivery and efficacy (205). Ongoing research aims to expand the range of cancers treatable with OVs and to refine combination regimens for maximum efficacy and safety.

## 7 Conclusions

Cancer is a heterogeneous and progressive disease that requires personalized treatment. Traditional therapies often lose effectiveness as tumors develop resistance, making the search for new combination therapies a significant challenge. Updated knowledge of virotherapy using oncolytic OVs, as summarized in this review, strongly suggests that shaping and modulating the tumor immune microenvironment may be a prerequisite for successful anti-cancer treatment. We anticipate that oncolytic viruses targeting or modulating the TIME may have beneficial therapeutic effects, particularly when used in combination with ICIs.

OVs have a dual effect on tumors: they destroy cancer cells and stimulate an antitumor immune response by inducing immune checkpoint inhibition and converting the TIME to an immunologically ‘hot’ state. However, OVs can also replicate in healthy cells, so targeting them specifically to cancer cells is essential. In this context, OVs carrying regulatory sequences (e.g., miRNA or tissue-specific promoters) help minimize the risk of uncontrolled viral spread, which is particularly important in immunocompromised cancer patients.

To improve ICD, immunostimulatory molecules are commonly integrated into OV genomes. Additionally, ICD induction can be enhanced by deleting viral genes that inhibit apoptosis. However, the synergistic effects of these modifications may vary significantly depending on the cancer type.

Light-activated oncolytic viruses could become indispensable and safe tools for translational studies, potentially accelerating the clinical development of novel anti-cancer therapies. Nevertheless, several intrinsic properties of optogenetic tools–such as background leakage, stability, biodistribution, and potential toxicity–remain significant barriers to future clinical application.

To summarize recent advances in virotherapeutic approaches, the main focus of translational research is on retargeting oncolytic viruses to bind specifically to cancer cells (using genetic or biochemical methods), enhancing the ability of viruses to induce ICD by altering the tumor microenvironment, and minimizing the risk of uncontrolled viral replication at off-target sites.

Despite the versatility of OV treatments, combined approaches involving chemotherapy, radiotherapy, and immunotherapy are likely to be the most effective and clinically applicable, while novel oncolytic-based therapeutics continue to undergo clinical trials.
